# Overload-based cascades on multiplex networks and effects of inter-similarity

**DOI:** 10.1371/journal.pone.0189624

**Published:** 2017-12-18

**Authors:** Dong Zhou, Ahmed Elmokashfi

**Affiliations:** Simula Research Laboratory, 1325 Lysaker, Norway; Beihang University, CHINA

## Abstract

Although cascading failures caused by overload on interdependent/interconnected networks have been studied in the recent years, the effect of overlapping links (inter-similarity) on robustness to such cascades in coupled networks is not well understood. This is an important issue since shared links exist in many real-world coupled networks. In this paper, we propose a new model for load-based cascading failures in multiplex networks. We leverage it to compare different network structures, coupling schemes, and overload rules. More importantly, we systematically investigate the impact of inter-similarity on the robustness of the whole system under an initial intentional attack. Surprisingly, we find that inter-similarity can have a negative impact on robustness to overload cascades. To the best of our knowledge, we are the first to report the competition between the positive and the negative impacts of overlapping links on the robustness of coupled networks. These results provide useful suggestions for designing robust coupled traffic systems.

## Introduction

Overloads can cause cascading failures in various critical infrastructures including power grids, transportation systems, and telecommunication networks. Therefore, in the past decade, modeling overload-based cascading failures in complex networks has attracted extensive interest [1–14]. One of the earliest models of overload-based cascades in networks was proposed by Motter and Lai [1]. They define the load on nodes using the betweenness centrality, and the tolerance capacity of each node is assumed to be proportional to its initial load. After their pioneering work, many extensions have been proposed [2–7], as well as other models of overload and traffic congestions [8–14].

Although previous studies on cascading failures based on overload have strongly deepened our understandings of the robustness of systems with traffic flows, most of them focused on single isolated networks. In fact, since most realistic complex systems are not isolated, but coupled in different ways, coupled networks, including interdependent networks, multiplex networks and interconnected networks, have become the most attractive fields in network science since 2010 [15–17]. Therefore, there is an need to understand overload-based cascades in coupled networks.

In this direction, several models have been developed, using Motter and Lai’s overload model or other overload definitions in interdependent networks [18–22] or interconnected networks [23–27]. For example, Tan *et al*. applied Motter’s model to interconnected scale-free networks under intentional attacks, and they found that there is an optimal fraction of interconnectivity links, which varies with different coupling rules [23]. Xia *et al*. presented a similar model but with capacity redundancy disparity, where increasing the capacity on one side may lead to less robust systems [25]. Further, Hong *et al*. presented a model of cascading failures on interdependent networks including overload-based failures [19]. However, until now, there are no studies of overload-based cascades on multiplex networks with one set of nodes but different types of links. More importantly, none of the previous models discussed the effects of overlapping links (inter-similarity) on the robustness of multiplex networks to overloads. Actually, the influence of inter-similarity is key to understanding the robustness of coupled systems with traffic flows, since many real-world coupled systems have a certain fraction of overlapping links.

In this paper, we propose a general model of overload-based cascading failures in multiplex networks, and study the effect of inter-similarity. Each node has different load values, as well as different tolerance capacities in different layers of the multiplex network. Three different coupling schemes and three overload rules are compared. Interestingly, we find that sometimes inter-similarity does not only increase assortativity, but can also have a negative impact on the robustness of the multiplex network to overload cascades. This is the first time both the good and the bad effects of inter-similarity on the robustness of coupled networks are reported.

## Materials and methods

### Network model

**Fig 1:**
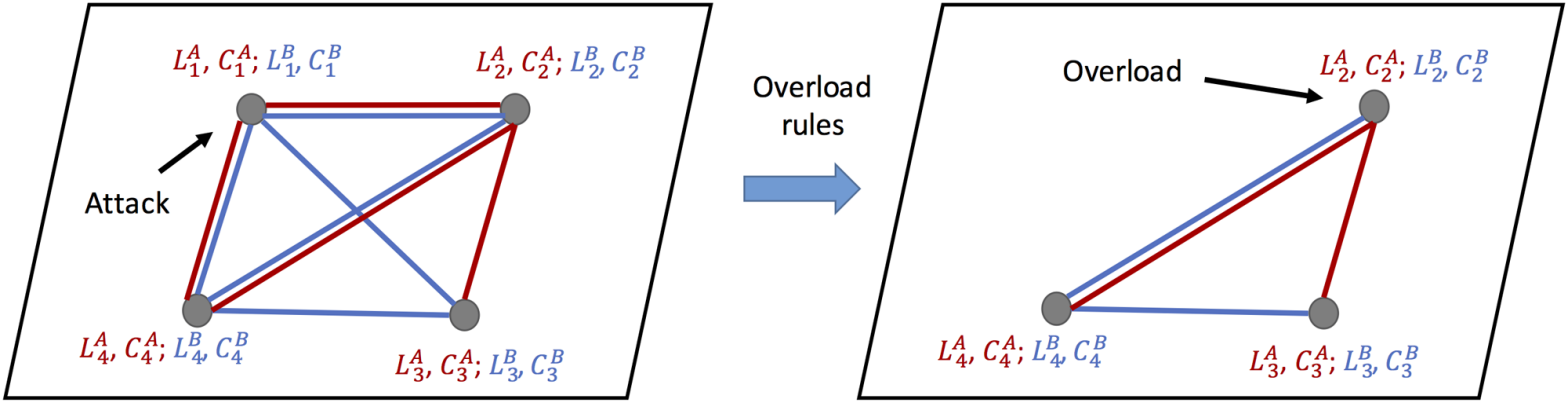
A schematic of our model of overload-based cascading failures on multiplex networks. The system of multiplex networks is composed of two layers *A* and *B* (red and blue links respectively), which share the same set of nodes. At each time step *t* ≥ 0, each node has two load values $L_{i}^{A}\left(t\right)$ and $L_{i}^{B}\left(t\right)$, defined as the its betweenness centrality in each layer. Each node also has two fixed capacities, defined as $C_{i}^{A}=\left(1+\alpha_{A}\right)\cdot L_{i}^{A}\left(0\right)$ and $C_{i}^{B}=\left(1+\alpha_{B}\right)\cdot L_{i}^{B}\left(0\right)$. At the beginning, the node with the largest total load $L_{i}^{A}\left(0\right)+L_{i}^{B}\left(0\right)$ is attacked. This leads to the redistribution of loads among the remaining nodes. At the next time step, according to some pre-defined overload rule, some other nodes will also fail due to overload. This causes a cascading process of overload failures, until no more overload happens.

In this paper, we aim to generalize the Motter and Lai’s overload based cascade model to multiplex networks. To this end, we consider a multiplex network composed of two single-layer networks *A* and *B* (depicted in Fig 1). Networks *A* and *B* share the same set of *N* nodes, but they can have different sets of links. Hence, the constructed multiplex network includes two types of interactions between nodes.

In this work, two types of network models have been used: Erdős-Rényi (ER) model and scale-free (SF) networks using Barabási–Albert (BA) model, which are homogeneous networks and heterogeneous random networks respectively. We also consider the following three types of coupling approaches:

1. **Random coupling:** Each node in network *A* is combined with a randomly selected node in network *B* one by one, until all nodes in networks *A* (*B*) have been one-to-one coupled with nodes in network *B* (*A*).
2. **Assortative coupling:** Nodes on each layer are sorted according to their betweenness (load) values. Then the node with the largest load in network *A* is merged with the node with the largest load in network *B*. The node with the second largest load in *A* is merged with its counterpart in *B*, and so on, until all nodes in *A* (*B*) have been coupled with their counterparts in *B* (*A*).
3. **Disassortative couplings:** Nodes in each layer are sorted according to their betweenness (load) values. The node with the largest load in network *A* is merged with the node with the smallest load in network *B*. The node with the second largest load in *A* is merged with the one with the second smallest load in *B*, and so on, until all nodes in *A* (*B*) have been coupled with their counterparts in *B* (*A*).

These three coupling approaches are considered in this work in order to systematically study the effects of assortative coupling by comparing with random or disassortative coupling. This is important, because we would like to verify whether the frequently observed assortative coupling in real-world coupled transport systems is really a good choice.

### Load-based cascades on multiplex networks

To model overload-based cascading failures on multiplex networks, we consider multiplex networks with two ER networks or two SF networks with the same size (number of nodes *N*), coupled following one of the above mentioned 3 coupling methods. We assume that initially (at time step *t* = 0) each node *i* = 1, ⋯, *N* has two load values: $L_{i}^{A}\left(0\right)$, $L_{i}^{B}\left(0\right)$, defined as the betweenness centrality values of node *i* in two layers *A* and *B* respectively. Each node *i* also has two capacity values: $C_{i}^{A}$ and $C_{i}^{B}$, defined as $C_{i}^{A}=\left(1+\alpha_{A}\right)\cdot L_{i}^{A}\left(0\right)$, and $C_{i}^{B}=\left(1+\alpha_{B}\right)\cdot L_{i}^{B}\left(0\right)$. The parameters *α*_*A*_ and *α*_*B*_ capture the load tolerance in different layers.

Initially, the node with the largest total load $L_{i}^{A}\left(0\right)+L_{i}^{B}\left(0\right)$ is attacked, and removed from both layers *A* and *B*. This leads to a redistribution of loads to the remaining nodes in both layers at *t* = 1: all the remaining nodes have new load values, $L_{i}^{A}\left(t\right)$ and $L_{i}^{B}\left(t\right)$, defined as the betweenness values in the remaining two layers.

In our model, the following three overload rules are defined:

1. **“OR rule”:** Node *i* fails at time step *t*, if $L_{i}^{A}\left(t\right)\geq C_{i}^{A}$ or $L_{i}^{B}\left(t\right)\geq C_{i}^{B}$.
2. **“SUM rule”:** Node *i* fails at time step *t*, if $L_{i}^{A}\left(t\right)+L_{i}^{B}\left(t\right)\geq C_{i}^{A}+C_{i}^{B}$.
3. **“AND rule”:** Node *i* fails at time step *t*, if $L_{i}^{A}\left(t\right)\geq C_{i}^{A}$ and $L_{i}^{B}\left(t\right)\geq C_{i}^{B}$.

According to the defined overload rule, at each time step *t* > 0, some nodes will fail due to overload, which leads to further redistributions of loads. This cascading process of failures will cease when no more overload happens. Following Motter and Lai’s model, we use the relative mutual giant component size to measure the robustness of the system, *G*, defined as the ratio of the size of the largest mutually connected component at the final state, and that after the initial attack (See Fig 1). We mainly aim to study the relationship between *G* and capacity tolerance parameters *α*_*A*_, *α*_*B*_, and to test the effects of network structures and coupling schemes.

## Results

### Basic model results

In this section, we show how network structures and assortativity affect the system robustness. Two types of network models are used here: random networks using Erdős-Rényi (ER) model and scale-free (SF) networks using Barabási–Albert (BA) model, which are homogeneous and heterogeneous networks respectively. We keep the system size as *N* = 500, and the mean degree of each layer as *k*_*A*_ = *k*_*B*_ = 6, for either coupled ER networks or coupled SF networks. We evaluate the system robustness under the three coupling schemes and the three overload rules described in the Methods section above.

**Fig 2:**
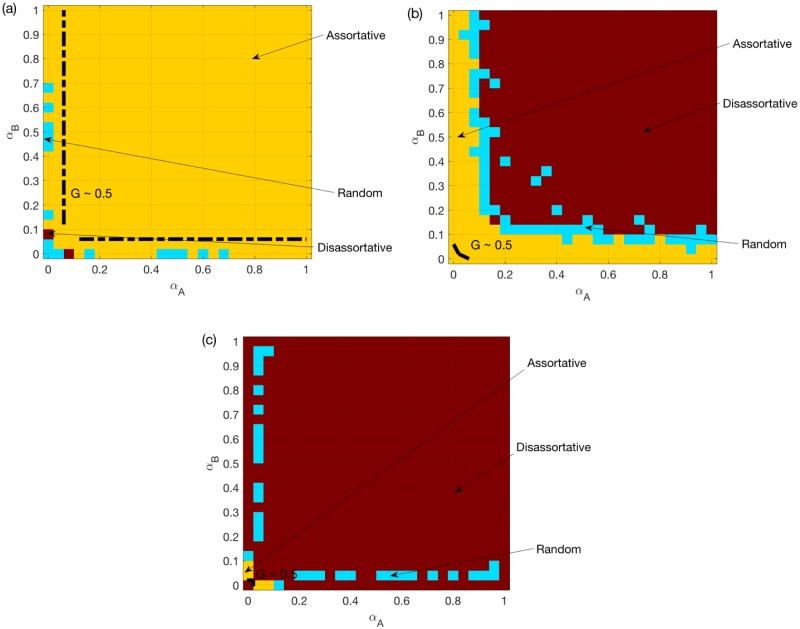
Best choice among random/assortative/disassortative coupling schemes for two ER networks. (a) OR rule. (b) SUM rule. (c) AND rule. *N* = 500, *k*_*A*_ = *k*_*B*_ = 6, averaged over *M* = 50 realizations. The color for certain *α*_*A*_ and *α*_*B*_ values indicates the coupling scheme with the largest mean *G* value. Since the largest mean *G* value (among the three coupling schemes) increases in general for larger *α*_*A*_ and *α*_*B*_, a dashed line is added to each sub-figure to indicate the location where the best mean *G* reaches 0.5.

Fig 2A shows the best choice (with the largest *G*) among random, assortative and disassortative coupling schemes for different *α*_*A*_, *α*_*B*_ values, for two ER networks under the OR rule. Note that in general the mean *G* increases from 0 to 1 as *α*_*A*_ or *α*_*B*_ increases, and the impacts of *α*_*A*_ and *α*_*B*_ on the model are symmetric. The dashed line is added where the mean *G* reaches 0.5, which indicates that the system is during the process of a transition between a congested state and a congestion-free state. The same dashed line will be included in all following figures. We find that, for two ER networks under the OR rule, assortative coupling is better in most cases.

Fig 2B and 2C shows the same as Fig 2A but under the SUM/AND rules. We find that, for two ER networks under the OR/SUM/AND rules, assortative coupling is always good when the system is during the transition process. Assortativity is no longer better when the traffic system is already efficient i.e. for larger *α*_*A*_, *α*_*B*_.

**Fig 3:**
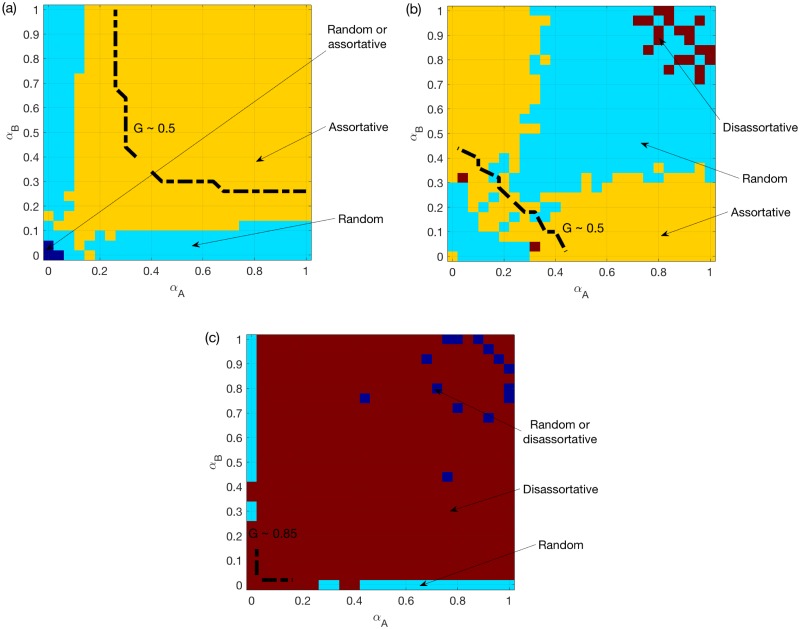
Best choice among random/assortative/disassortative coupling schemes for two SF networks. (a) OR rule. (b) SUM rule. (c) AND rule. *N* = 500, *k*_*A*_ = *k*_*B*_ = 6, averaged over *M* = 50 realizations. The color for certain *α*_*A*_ and *α*_*B*_ values indicates the coupling scheme with the largest mean *G* value. A dashed line is added to each sub-figure to indicate the location where the best mean *G* reaches 0.5, or 0.85 for (c).

Fig 3A–3C shows the same as Fig 2, but for two SF networks. We find that assortativity is still good for the system under the OR rule, but not under the AND rule. As shown in Fig 3B, for two SF networks under the SUM rule, random coupling is the best for most symmetric systems i.e. those with small differences between *α*_*A*_ and *α*_*B*_. However, for non-symmetric systems, i.e. systems with large differences between *α*_*A*_ and *α*_*B*_, assortative coupling is the best. When both *α*_*A*_ and *α*_*B*_ are around 0.2, the best is a combination of random coupling and assortative coupling.

We next provide probable explanations for these observations. *Firstly*, for symmetric systems, assortativity is good for two SFs under the OR rule, since it reduces the chance of having an overload on one of the two layers. Similarly, assortativity is bad for two SFs under the AND rule. This also applies to two ERs, although the observed effects will be weakened due to a much more homogeneous capacities.

*Secondly*, we consider non-symmetric systems e.g. where *α*_*A*_ < <*α*_*B*_. Under the OR rule, because of the much larger nodes’ capacities in *B*, the overload rule approximately reduces to “$L_{i}^{A}\left(t\right)\geq C_{i}^{A}$”. Therefore, the advantage of the assortative coupling will disappear, especially for two SFs (see Fig 3A). Under the AND rule, even when $C_{i}^{A}<<C_{i}^{B}$, failures still happen when the two layers experience overload simultaneously. Therefore, the disassortative coupling is better in general.

*Finally*, we focus on the SUM rule. For two SFs, the assortative coupling brings more heterogeneity for both the total load and capacity. Therefore, assortativity has a much weaker and more complicated impact under the SUM rule compared to the other two rules. The assortative coupling can negatively affect the system, because attacking the node with the largest total load usually translates into attacking hub nodes in both layers. This explains why the random coupling is better than either assortative or disassortative coupling for symmetric systems see Fig 3B. For coupled ERs, such intentional attacks have minimal impact. In fact, the assortative coupling leads to broader total loads and capacities distributions, when *α*_*A*_ and *α*_*B*_ are small such that the total capacities are smaller than the total loads on average. This increases the chance of having $L_{i}^{A}\left(t\right)+L_{i}^{B}\left(t\right)<C_{i}^{A}+C_{i}^{B}$, which explains why the best choice changes from assortative to disassortative coupling in Fig 2B.

### Impact of inter-similarity

In this section, we study the effect of inter-similarity on cascading failures in multiplex networks. To this end, we vary the percentage of overlapping links, 0% ≤ *r* ≤ 100%, in the multiplex ER or SF networks. Note that here *r* = 0% is the same as random coupling.

For coupled ERs, to modify the overlap ratio *r* and keep the mean degree *k*_*A*_ = *k*_*B*_ = 6 in each layer, we generate three ER networks *A*′, *B*′ an *C* with mean degree 6(1 − *r*), 6(1 − *r*) and 6*r*, independently. Then we obtain two ER networks *A* and *B* with a ratio *r* of overlapping links by taking the sum of the adjacency matrices: *A* = *A*′ + *C* and *B* = *B*′ + *C*. The multiplex system is then constructed by merging the nodes from the two layers one-to-one without node shuffling.

For coupled SFs, we firstly generate two identical SF networks, *A* and *B*, with mean degree *k*_*A*_ = *k*_*B*_ = 6, and we sort the nodes in the two networks in the same order. Then we randomly select a fraction *p* of nodes from network *B* and randomly shuffle their order. Using this new node order, all nodes in the two layers are merged to construct the multiplex networks. Note that the node shuffling keeps the single-layer network structures but leads to a fraction *r* ≈ (1 − *p*)^2^ of overlapping links. (Notice that after randomly shuffling a fraction *p* of nodes, the number of remaining overlapping links is then [(1 − *p*)*N*]([(1 − *p*)*N*] − 1)/2, which is approximately a fraction (1 − *p*)^2^ of the total number of links *N*(*N* − 1)/2.) Therefore, to finally obtain a fraction *r* of overlapping links, we use $p=1-\sqrt{r}$.

**Fig 4:**
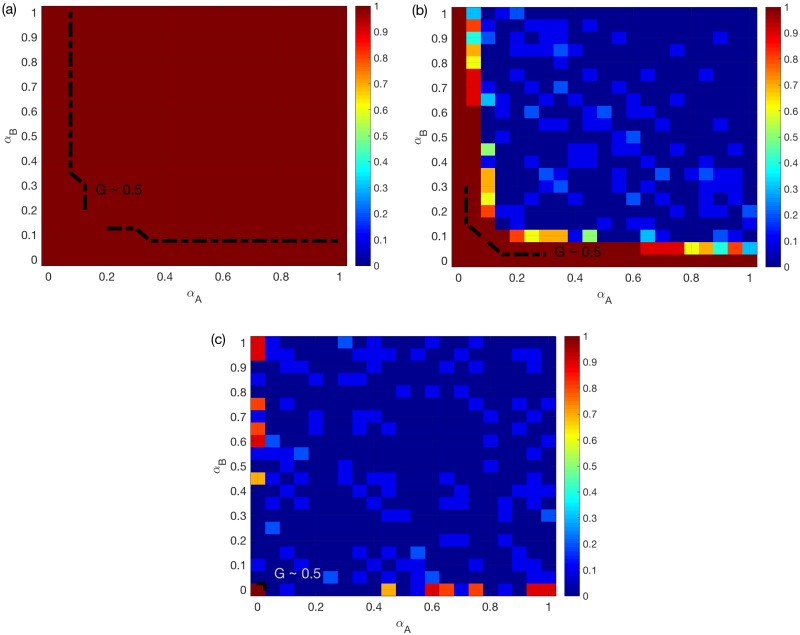
Best choice of overlap ratio *r* for two ER networks. (a) OR rule. (b) SUM rule. (c) AND rule. *N* = 500, *k*_*A*_ = *k*_*B*_ = 6, averaged over *M* = 90 realizations. The color for certain *α*_*A*_ and *α*_*B*_ values indicates the optimal *r* that leads to the largest mean *G* value. A dashed line is added to each sub-figure to indicate the location where the best mean *G* reaches 0.5.

We first illustrate the effects of inter-similarity for two ER networks under different overload rules. Fig 4A–4C shows the best choice of the overlap ratio *r* for the OR/SUM/AND rules respectively. A dashed line indicating mean *G* around 0.5 is added again. We find that *r* = 100% and *r* ≈ 0% overlap ratios are the best under the OR rule and the AND rule. Under the SUM rule, full overlapping is the best when the system is during the transition process, while 0% becomes better when both *α*_*A*_ and *α*_*B*_ are larger. These patterns are similar to the effect of assortativity.

**Fig 5:**
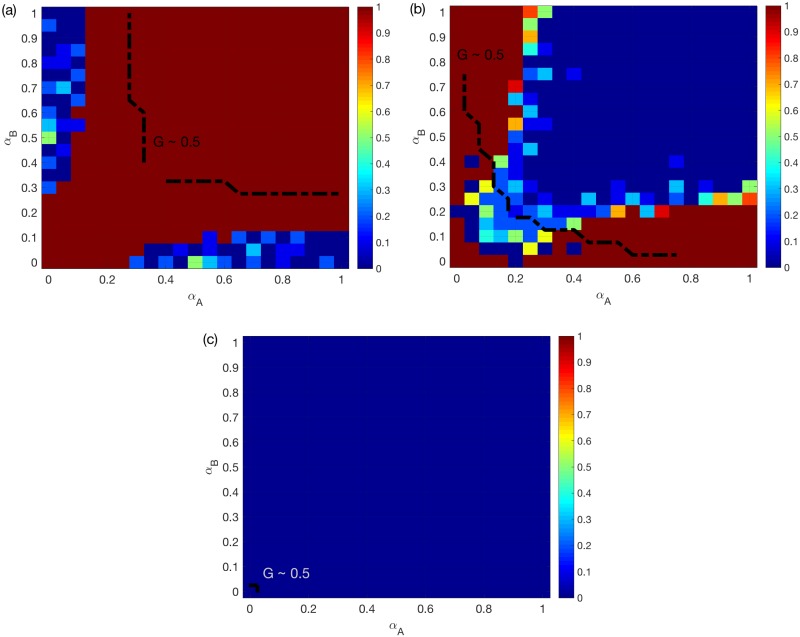
Best choice of overlap ratio *r* for two SF networks. (a) OR rule. (b) SUM rule. (c) AND rule. *N* = 500, *k*_*A*_ = *k*_*B*_ = 6, averaged over *M* = 90 realizations. The color for certain *α*_*A*_ and *α*_*B*_ values indicates the optimal *r* that leads to the largest mean *G* value. A dashed line is added to each sub-figure to indicate the location where the best mean *G* reaches 0.5.

For two SFs, as shown in Fig 5A–5C, the best overlap ratios also closely match the effect of assortativity. One interesting finding here is that, according to Fig 5B, when *α*_*A*_ and *α*_*B*_ increase the best choice of overlap ratio can have continuous changes from 1 to 0, especially when the system is during the transition process. Compared to Fig 3B, we note that this continuous change happens within the region where assortativity is good for the system. This is a noteworthy observation, because it shows that although assortative coupling is good for avoiding cascades, the optimal overlap ratio is not a trivial 100% or 0% as in the other cases. This indicates that SF multiplex networks need to be designed carefully when operating under the SUM rule. Note also that *α*_*A*_ and *α*_*B*_ values for real systems mostly lie in the region with continuos change. This is the first time inter-similarity is found to have a non-trivial impact on the robustness of multiplex networks to overload compared to assortative coupling.

### A holistic view

In this section, we provide a holistic view by comparing various coupling schemes to coupling with inter-similarity. This gives a better idea about all available options for improving multiplex networks resilience to overload cascades.

**Fig 6:**
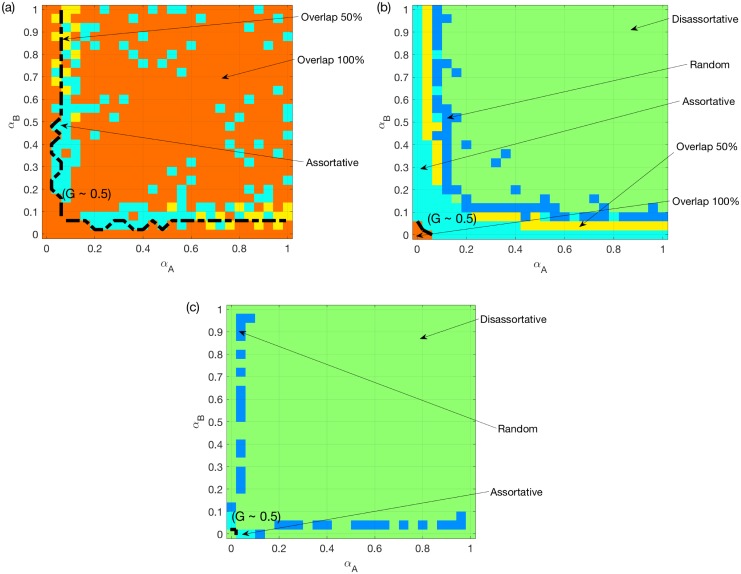
Best choice of coupling schemes for two ER networks. (a) OR rule. (b) SUM rule. (c) AND rule. *N* = 500, *k*_*A*_ = *k*_*B*_ = 6, averaged over *M* = 50 realizations. The color for certain *α*_*A*_ and *α*_*B*_ values indicates the best coupling scheme having the largest mean *G* value. A dashed line is added to each sub-figure to indicate the location where the best mean *G* reaches 0.5.

Firstly, we show, for two ER networks, the best choice among the following five coupling schemes: random coupling, assortative coupling, disassortative coupling, a 50% overlap, and a 100% overlap. Fig 6A and 6C show as expected that, under the OR or AND rule, a 100% overlap or disassortative coupling are the best coupling approaches in most cases. Fig 6B, however, shows the presence of several regions under the SUM rule. The best choice changes from 100% overlap, assortative coupling to random/disassortative coupling. Interestingly, the region where the best option is assortative coupling corresponds to the region where 100% is the best overlap ratio in Fig 4B. Accordingly, 100% overlap is worse than assortative coupling in this region, although it leads to full assortativity. The same holds under the OR rule for highly non-symmetric cases and under the AND rule around the transition region. This result is surprising since it shows that inter-similarity not only brings coupling assortativity, but can also have both positive and negative impact (good and bad sides) on multiplex networks resilience to overload cascades.

**Fig 7:**
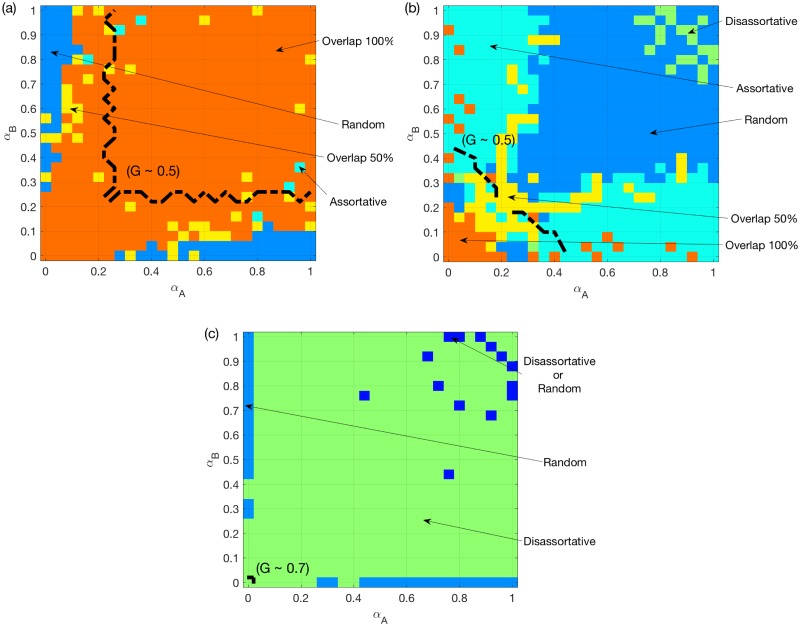
Best choice of coupling schemes for two SF networks. (a) OR rule. (b) SUM rule. (c) AND rule. *N* = 500, *k*_*A*_ = *k*_*B*_ = 6, averaged over *M* = 50 realizations. The color for certain *α*_*A*_ and *α*_*B*_ values indicates the best coupling scheme having the largest mean *G* value. A dashed line is added to each sub-figure to indicate the location where the best mean *G* reaches 0.5, and 0.7 for (c).

Secondly, we perform the same comparison for a multiplex network of two SF networks. Fig 7 presents the corresponding results. Fig 7A and 7C show that, under the OR/AND rules, the best options are similar to what we have seen in Figs 5A and 3C. For the SUM rule, Fig 7B shows that, similar to Fig 5B, a 50% overlap remains the best option in the region where both *α*_*A*_ and *α*_*B*_ are around 0.2. Interestingly, we find that, for non-symmetric systems, a full overlap is worse than the assortative coupling.

The above interesting finding suggests that a 100% overlap, besides bringing assortativity, can have a negative impact on the traffic system on multiplex networks. This is especially evident for two ER networks under the SUM/AND rules. In order to interpret this, we investigate the differences between the assortative coupling and the 100% overlap. For both of them, nodes with larger loads/capacities in *A* are coupled with nodes with larger loads/capacities in *B*. The major difference is that for 100% overlap the two layers are identical. This translates into a higher likelihood, compared to assortative coupling, for meeting requirements of the SUM or AND rules, but not the OR rule. Therefore, inter-similarity is more likely to result in overload, and also increases assortativity. In regions where assortativity is good, there will be a competition between these two effects. As a result, we observe the non-integer best overlap ratio in Fig 5B i.e. the optimality of assortative coupling (instead of full overlap) in some regions in Figs 6A–6C and 7B.

**Fig 8:**
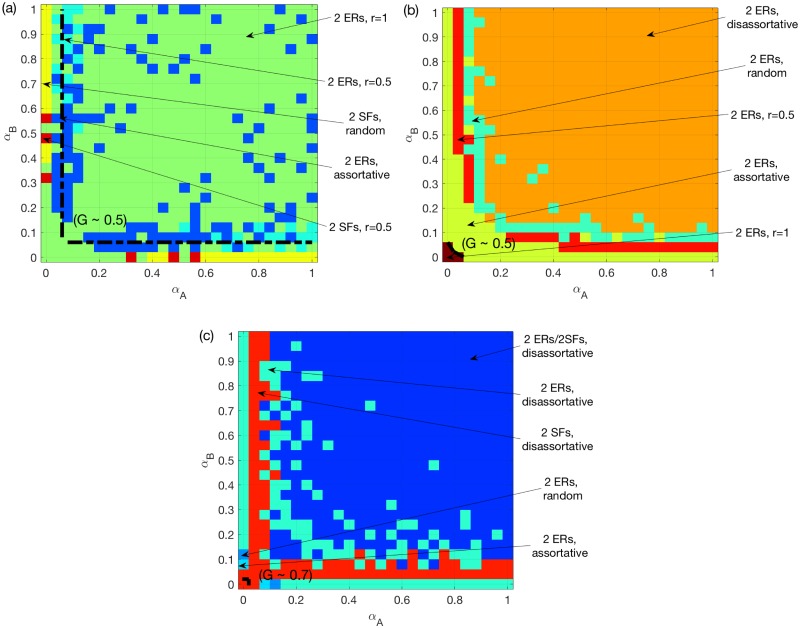
Best choice of network structures and coupling schemes. (a) OR rule. (b) SUM rule. (c) AND rule. *N* = 500, *k*_*A*_ = *k*_*B*_ = 6, averaged over *M* = 50 realizations. The color for certain *α*_*A*_ and *α*_*B*_ values indicates the best type of networks and coupling scheme having the largest mean *G* value. A dashed line is added to each sub-figure to indicate the location where the best mean *G* reaches 0.5, and 0.7 for (c).

Finally, we compare the resilience between homogeneous and heterogeneous multiplex networks to overload cascades. To this end, we show in Fig 8A–8C the best choice among the following 10 options: 2 choices of network structures (two ERs or two SFs) and 5 choices of coupling schemes (random/assortative/disassortative coupling, 50% and 100% overlap), under different overload rules respectively. We find that in most cases the optimal choice for two ER networks is better than the best choice for the two SFs. The main exceptions are the regions where one of *α*_*A*_, *α*_*B*_ is very small under the OR or the AND rule. This suggests that using homogeneous networks can be one way to improve the robustness of multiplex networks to overload cascades.

### Effects of network sizes

**Fig 9:**
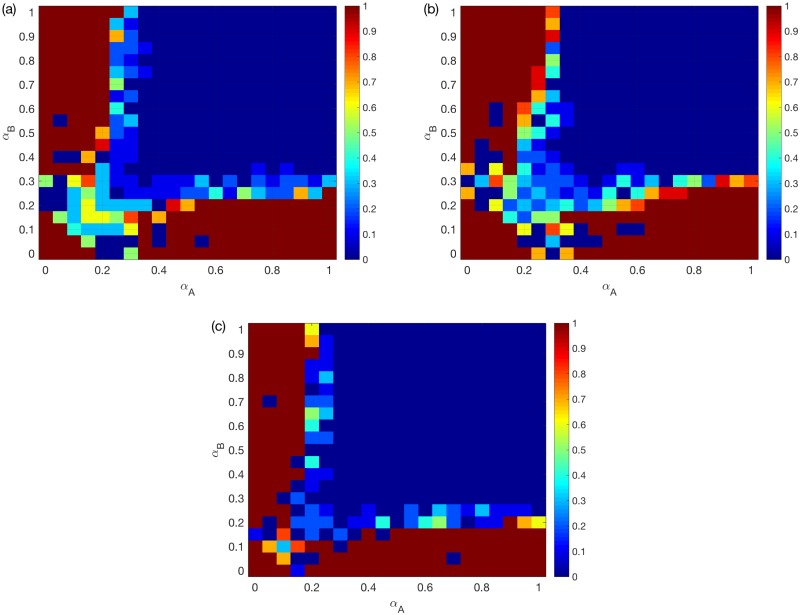
Best choice of overlap ratio *r* for two SF networks, SUM rule. (a) *N* = 1000, averaged over *M* = 30 realizations. (b) *N* = 2000, averaged over *M* = 30 realizations. (c) *N* = 3000, averaged over *M* = 5 realizations *k*_*A*_ = *k*_*B*_ = 6. The color for different *α*_*A*_ and *α*_*B*_ values indicates the optimal *r* that leads to the largest mean *G* value.

All the above model results are obtained based on multiplex networks with *N* = 500. This smaller network size is used through our work for reducing computational complexity. Moreover, many real-world coupled traffic systems also have smaller network sizes. In the following, we investigate whether increasing *N* qualitatively impact our results. To this end, we recompute the results in Fig 5B, which is one of the key findings in this work, for larger networks. Fig 9A and 9B shows the best choice of overlap ratio for coupled SF networks with *N* = 1000 and *N* = 2000, respectively, averaged over *M* = 30 realizations. Fig 9C further shows the case with *N* = 3000, but with *M* = 5 realizations. We find that the same pattern with Fig 5B can be also found here: the optimal overlap ratio still can be neither 0 or 1 at around *α*_*A*_ = *α*_*B*_ ∼ 0.2. This suggests that the major findings of our work continue to hold for networks with up to thousands of nodes.

Although it is not easy to show the simulation results for further larger systems, we can claim that the discovered negative impact of inter-similarity still hold for large networks, since it is mainly related to the nature of the SUM and AND overload rules. The detailed effects of *N* on the competition between the positive and negative impacts of inter-similarity can be studied in future work.

### Realistic coupled communication networks

In the previous subsections, the model results have been presented on coupled random network models. To relate our model to real world coupled systems with traffic loads, we apply the model on realistic Internet topologies at the autonomous system (AS) level, using IPv4 and IPv6 respectively. The Internet topology consists of a large number of interconnected ASes. These ASes use devices called routers to facilitate traffic exchange between them. Currently, there are two addressing schemes in the Internet IPv4 and IPv6, resulting in two partially overlapping AS-level topologies. IPv6 is currently maturing which makes its graph smaller. In most cases, the same router is used to forward IPv4 and IPv6 traffic which may result in overloads. To obtain the network structures of IPv4 and IPv6, we use the CAIDA datasets of AS links (“http://www.caida.org/data/active/ipv4_routed_topology_aslinks_dataset.xml” for IPv4 and “http://www.caida.org/data/active/ipv6_aslinks_dataset.xml” for IPv6). We consider the topologies collected in January of 4 different years (2011, 2013, 2015 and 2017) as different stages, and we collect all the AS nodes and direct AS links from the CAIDA records in each stage to construct the whole network. Finally, for each stage, we build the multiplex IPv4/IPv6 networks by merging the same AS nodes in two layers, and only focusing on the shared nodes and the IPv4/IPv6 links among them.

**Fig 10:**
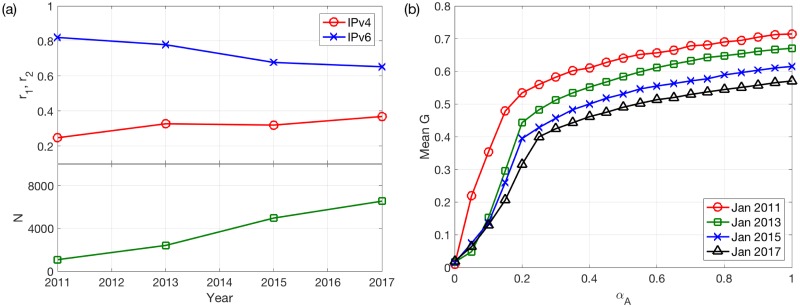
Results on real-world coupled IPv4/IPv6 networks. (a) Fraction of overlapping links, *r*_1_ and *r*_2_ and the system size *N* in the multiplex IPv4/IPv6 networks in January of different years. *N* = 1086, 2416, 4974, and 6550, respectively. (b) Mean *G* as a function of *α*_*A*_ in the model with realistic IPv4/IPv6 networks and the SUM rule. January in different years, averaged over *M* = 30 realizations.

Fig 10A shows how the fractions of overlapping links in IPv4 and IPv6 networks, *r*_1_ and *r*_2_, and the number of nodes *N*, change over time. We find that the common links account for a larger fraction in the IPv6 topology, since there are much more IPv4 links in these stages compared to IPv6 links. Moreover, the fraction of common links in the IPv6 network decreases with time, while that in the IPv4 network is roughly fixed at around 0.35.

We run our model using the SUM rule on the constructed multiplex IPv4/IPv6 networks. We choose the SUM rule, because in the realistic IPv4 and IPv6 networks, the load of one node can be shared by the two layers. Moreover, we mainly focus on the cases with *α*_*A*_ = *α*_*B*_, since the IPv4 and IPv6 are expected to have similar tolerance capacities. The model results are shown in Fig 10B. We find that the earlier stages are more robust to overload cascades. Considering the heterogeneous structures of IPv4/IPv6 networks, this result (smaller fractions of common links corresponding to less efficient traffic systems) can be related to the previous model results as shown in Fig 5B, where the best overlapping ratio changes from 1 to smaller values during the transition process. Our results suggest that it is necessary to pay attention to the fraction of common links between IPv4 and IPv6 for obtaining more reliable telecommunication networks. However, it is still hard to determine whether the change of the overlapping ratio is the main reason for the observed decrease in robustness, since the two layers have different amounts of links, and the system size also varies with time, which differ from our model.

## Discussion

In this work, we develop a new model of overload-based cascades (following Motter and Lai’s model of overload) on multiplex networks, and we investigate the impact of inter-similarity. This is not the first time that cascading failures caused by overload on multiplex networks are investigated. K. Lee et al., have studied the sandpile model on multiplex networks in 2012 [28]. They compared duplex ER networks and SF networks, as well as overload rules (like our OR rule and AND rule). However, the sandpile model mainly focuses on generating power-law failure size distributions at criticality, rather than the efficiency of traffic flow or robustness to overload cascades, which we focus on.

We study the robustness of multiplex networks to overload cascade, which is a different way of modeling overload on coupled networks as oppose to previous models using interconnected or interdependent networks. We assume different types of flows in the different layers and three overload rules: OR, AND, and SUM. Under the OR rule, our model is similar to previous fully interdependent networks models. The main difference is that we consider failures caused by single-layer overload and interdependencies at each step. Percolation, however, is only considered at the end of the cascade. Moreover, our model with the AND rule can be applied to coupled traffic systems where an overloaded node in one layer can survive if it is not overloaded in the other layer. The SUM rule lies between the OR and the AND rules, where the load can be shared by the two layers.

We compare homogeneous and heterogeneous networks, and random/assortative/disassortative coupling schemes. We further study the effects of inter-similarity by finding the best overlap ratio. One of the most interesting findings is that for two SF networks under the SUM rule, the best overlap ratio decreases from 1 to 0 in a continues fashion when *α*_*A*_ or *α*_*B*_ are around 0.2. This is important since many critical coupled systems with traffic flow have heterogeneous structures and not very large capacity tolerance. This finding provides suggestions for the optimal fraction of overlapping links for the real world multiplex systems. Another surprising finding is that for two ERs under the SUM/AND rules, during the transition process, a full overlap is worse than assortative coupling, although assortativity is good for the system. To explain these findings, we for the first time clarify both the positive and negative potential impacts of inter-similarity simultaneously on the robustness of multiplex networks. Note that in previous works, inter-similarity has been proved to be able to improve the system robustness in percolation of interdependent or multiplex networks [29–31]. We also find that homogenous multiplex networks i.e. two ERs are more robust to overload cascades compared to heterogeneous two SF multiplex networks. This supplements the previous understanding that interdependent SF networks are more vulnerable than interdependent ER networks [15]. We have verified that the major model results hold for *N* as large as around 3000. More detailed effects of network sizes can be investigated in future work. We also plan to explore overload-based cascades on systems with more than two sub-networks and effects of inter-similarity in our future work.

In summary, we have proposed a model for studying overload-based cascading failures in multiplex networks. We have systematically explored the effect of inter-similarity and assortativity coupling. A key finding is that inter-similarity can have both positive and negative impact. We have explained our surprising findings and discussed their potential applications to real world multiplex networks.
